# Supplementary material for: Modeling glioblastoma heterogeneity as a dynamic network of cell states
Source: Mol Syst Biol. 2021 Sep 16;17(9):e10105. doi: 10.15252/msb.202010105 (PMC8444284; doi:10.15252/msb.202010105)
Supplement: Supplementary file 5 — Source Data for Figure 3 [file MSB-17-e10105-s001.zip › Figure3A_sourcedata/GSEA_3065/nosigdb_state6.GseaPreranked.1622718443993/RICHARDS_NOWAKOWSKI_SCIENCE_2017_IN_CTX_MGE2_UPREG.html]

Details for gene set RICHARDS\_NOWAKOWSKI\_SCIENCE\_2017\_IN\_CTX\_MGE2\_UPREG[GSEA]

|  || Dataset | state6 |
| Phenotype | NoPhenotypeAvailable |
| Upregulated in class | na\_pos |
| GeneSet | RICHARDS\_NOWAKOWSKI\_SCIENCE\_2017\_IN\_CTX\_MGE2\_UPREG |
| Enrichment Score (ES) | 0.29285416 |
| Normalized Enrichment Score (NES) | 1.373199 |
| Nominal p-value | 0.015384615 |
| FDR q-value | 0.10555477 |
| FWER p-Value | 0.946 |
Table: GSEA Results Summary

  

Fig 1: Enrichment plot: RICHARDS\_NOWAKOWSKI\_SCIENCE\_2017\_IN\_CTX\_MGE2\_UPREG      
 Profile of the Running ES Score & Positions of GeneSet Members on the Rank Ordered List

  

| PROBE | GENE SYMBOL | GENE\_TITLE | RANK IN GENE LIST | RANK METRIC SCORE | RUNNING ES | CORE ENRICHMENT || 1 | SOX4 |  |  | 3 | 0.375 | 0.0268 | Yes |
| 2 | NDUFC1 |  |  | 37 | 0.255 | 0.0414 | Yes |
| 3 | FHL1 |  |  | 61 | 0.236 | 0.0557 | Yes |
| 4 | SOX11 |  |  | 70 | 0.230 | 0.0715 | Yes |
| 5 | UQCR11 |  |  | 71 | 0.230 | 0.0881 | Yes |
| 6 | SNRPN |  |  | 115 | 0.206 | 0.0980 | Yes |
| 7 | TTC3 |  |  | 160 | 0.188 | 0.1064 | Yes |
| 8 | UQCRH |  |  | 208 | 0.174 | 0.1135 | Yes |
| 9 | UQCR10 |  |  | 257 | 0.161 | 0.1196 | Yes |
| 10 | SH3GLB1 |  |  | 260 | 0.161 | 0.1309 | Yes |
| 11 | CUX1 |  |  | 270 | 0.157 | 0.1413 | Yes |
| 12 | ARL4C |  |  | 276 | 0.156 | 0.1520 | Yes |
| 13 | BRK1 |  |  | 364 | 0.141 | 0.1520 | Yes |
| 14 | PDE4DIP |  |  | 392 | 0.137 | 0.1588 | Yes |
| 15 | CEP170 |  |  | 452 | 0.128 | 0.1612 | Yes |
| 16 | SVIL |  |  | 471 | 0.126 | 0.1682 | Yes |
| 17 | CREBBP |  |  | 484 | 0.125 | 0.1759 | Yes |
| 18 | FIS1 |  |  | 530 | 0.120 | 0.1793 | Yes |
| 19 | STAU1 |  |  | 538 | 0.119 | 0.1871 | Yes |
| 20 | PIP4K2B |  |  | 548 | 0.118 | 0.1946 | Yes |
| 21 | SATB1 |  |  | 566 | 0.117 | 0.2011 | Yes |
| 22 | HDAC9 |  |  | 574 | 0.116 | 0.2087 | Yes |
| 23 | TCEA1 |  |  | 576 | 0.116 | 0.2169 | Yes |
| 24 | BNIP2 |  |  | 617 | 0.112 | 0.2204 | Yes |
| 25 | MRPL21 |  |  | 620 | 0.112 | 0.2283 | Yes |
| 26 | ARID5B |  |  | 638 | 0.111 | 0.2344 | Yes |
| 27 | PSMF1 |  |  | 769 | 0.101 | 0.2265 | Yes |
| 28 | SLC29A4 |  |  | 780 | 0.100 | 0.2326 | Yes |
| 29 | QKI |  |  | 820 | 0.097 | 0.2350 | Yes |
| 30 | ATRX |  |  | 861 | 0.094 | 0.2372 | Yes |
| 31 | ATXN10 |  |  | 883 | 0.092 | 0.2414 | Yes |
| 32 | ENSA |  |  | 896 | 0.091 | 0.2466 | Yes |
| 33 | ARHGAP1 |  |  | 897 | 0.091 | 0.2532 | Yes |
| 34 | TPGS2 |  |  | 913 | 0.090 | 0.2580 | Yes |
| 35 | HTATSF1 |  |  | 927 | 0.090 | 0.2629 | Yes |
| 36 | HERC1 |  |  | 931 | 0.089 | 0.2690 | Yes |
| 37 | GID8 |  |  | 938 | 0.089 | 0.2748 | Yes |
| 38 | PFN2 |  |  | 942 | 0.089 | 0.2809 | Yes |
| 39 | PDHA1 |  |  | 977 | 0.087 | 0.2832 | Yes |
| 40 | TXLNA |  |  | 1105 | 0.080 | 0.2742 | Yes |
| 41 | BBX |  |  | 1139 | 0.078 | 0.2761 | Yes |
| 42 | MRPS21 |  |  | 1162 | 0.077 | 0.2791 | Yes |
| 43 | ZCRB1 |  |  | 1171 | 0.077 | 0.2837 | Yes |
| 44 | TCF4 |  |  | 1234 | 0.074 | 0.2819 | Yes |
| 45 | SNX27 |  |  | 1236 | 0.074 | 0.2871 | Yes |
| 46 | NIPSNAP1 |  |  | 1314 | 0.071 | 0.2833 | Yes |
| 47 | SLAIN1 |  |  | 1325 | 0.070 | 0.2872 | Yes |
| 48 | MRPS10 |  |  | 1334 | 0.070 | 0.2913 | Yes |
| 49 | PSIP1 |  |  | 1372 | 0.068 | 0.2920 | Yes |
| 50 | CCT7 |  |  | 1407 | 0.067 | 0.2929 | Yes |
| 51 | NXPH1 |  |  | 1558 | 0.062 | 0.2799 | No |
| 52 | ARRDC3 |  |  | 1579 | 0.061 | 0.2820 | No |
| 53 | SSU72 |  |  | 1617 | 0.060 | 0.2820 | No |
| 54 | ATCAY |  |  | 1635 | 0.059 | 0.2843 | No |
| 55 | RNF34 |  |  | 1699 | 0.057 | 0.2811 | No |
| 56 | GTF3C6 |  |  | 1703 | 0.057 | 0.2848 | No |
| 57 | JAKMIP2 |  |  | 1755 | 0.055 | 0.2829 | No |
| 58 | AP3M1 |  |  | 1766 | 0.055 | 0.2857 | No |
| 59 | MEF2C |  |  | 1785 | 0.054 | 0.2875 | No |
| 60 | BEX4 |  |  | 1823 | 0.053 | 0.2870 | No |
| 61 | ANAPC5 |  |  | 1859 | 0.052 | 0.2867 | No |
| 62 | UBXN2B |  |  | 1865 | 0.052 | 0.2898 | No |
| 63 | RASAL2 |  |  | 1966 | 0.049 | 0.2817 | No |
| 64 | ZC3H13 |  |  | 1968 | 0.049 | 0.2851 | No |
| 65 | EIF4G1 |  |  | 2046 | 0.046 | 0.2795 | No |
| 66 | OCIAD2 |  |  | 2061 | 0.046 | 0.2812 | No |
| 67 | UQCRB |  |  | 2152 | 0.043 | 0.2739 | No |
| 68 | CDC123 |  |  | 2157 | 0.043 | 0.2765 | No |
| 69 | PDCD5 |  |  | 2215 | 0.041 | 0.2728 | No |
| 70 | ANKRD13C |  |  | 2263 | 0.040 | 0.2702 | No |
| 71 | CDC42BPA |  |  | 2279 | 0.039 | 0.2713 | No |
| 72 | NF2 |  |  | 2298 | 0.039 | 0.2720 | No |
| 73 | BLOC1S1 |  |  | 2417 | 0.036 | 0.2609 | No |
| 74 | TNRC6C |  |  | 2450 | 0.035 | 0.2597 | No |
| 75 | SEPT7 |  |  | 2478 | 0.034 | 0.2590 | No |
| 76 | DDX6 |  |  | 2557 | 0.032 | 0.2522 | No |
| 77 | LSM3 |  |  | 2673 | 0.029 | 0.2410 | No |
| 78 | BUD31 |  |  | 2675 | 0.029 | 0.2430 | No |
| 79 | ZCCHC10 |  |  | 2716 | 0.029 | 0.2404 | No |
| 80 | ARHGEF7 |  |  | 2777 | 0.027 | 0.2354 | No |
| 81 | PSMD6 |  |  | 2779 | 0.027 | 0.2372 | No |
| 82 | UBQLN1 |  |  | 2845 | 0.026 | 0.2315 | No |
| 83 | PSMD14 |  |  | 2855 | 0.025 | 0.2323 | No |
| 84 | CCNG2 |  |  | 2949 | 0.023 | 0.2231 | No |
| 85 | NDUFB4 |  |  | 3026 | 0.021 | 0.2158 | No |
| 86 | ZBTB21 |  |  | 3143 | 0.019 | 0.2037 | No |
| 87 | HIP1R |  |  | 3156 | 0.019 | 0.2037 | No |
| 88 | SUGT1 |  |  | 3171 | 0.018 | 0.2034 | No |
| 89 | MAPT |  |  | 3232 | 0.017 | 0.1976 | No |
| 90 | TAOK1 |  |  | 3341 | 0.015 | 0.1861 | No |
| 91 | BRD2 |  |  | 3372 | 0.014 | 0.1836 | No |
| 92 | TNRC6B |  |  | 3434 | 0.012 | 0.1774 | No |
| 93 | PAK1IP1 |  |  | 3451 | 0.012 | 0.1764 | No |
| 94 | CDC5L |  |  | 3466 | 0.012 | 0.1757 | No |
| 95 | NARF |  |  | 3540 | 0.011 | 0.1679 | No |
| 96 | SMARCC1 |  |  | 3546 | 0.010 | 0.1681 | No |
| 97 | SPATS2L |  |  | 3639 | 0.008 | 0.1580 | No |
| 98 | THUMPD1 |  |  | 3657 | 0.008 | 0.1566 | No |
| 99 | EFR3B |  |  | 3683 | 0.007 | 0.1542 | No |
| 100 | DROSHA |  |  | 3760 | 0.006 | 0.1458 | No |
| 101 | MTSS1 |  |  | 3804 | 0.005 | 0.1411 | No |
| 102 | ZNF91 |  |  | 3825 | 0.004 | 0.1391 | No |
| 103 | RAD23B |  |  | 3871 | 0.003 | 0.1341 | No |
| 104 | ATRNL1 |  |  | 3976 | 0.001 | 0.1221 | No |
| 105 | ANKRD28 |  |  | 4017 | 0.000 | 0.1174 | No |
| 106 | NPAS3 |  |  | 4053 | -0.000 | 0.1134 | No |
| 107 | DDB1 |  |  | 4082 | -0.001 | 0.1102 | No |
| 108 | PPIP5K2 |  |  | 4084 | -0.001 | 0.1101 | No |
| 109 | DCAF8 |  |  | 4281 | -0.005 | 0.0877 | No |
| 110 | MYCBP2 |  |  | 4340 | -0.006 | 0.0814 | No |
| 111 | GNB5 |  |  | 4566 | -0.010 | 0.0560 | No |
| 112 | MINOS1 |  |  | 4665 | -0.012 | 0.0454 | No |
| 113 | ZEB2 |  |  | 4735 | -0.013 | 0.0384 | No |
| 114 | BRINP2 |  |  | 4802 | -0.014 | 0.0317 | No |
| 115 | TERF1 |  |  | 4804 | -0.014 | 0.0327 | No |
| 116 | GAD1 |  |  | 4858 | -0.016 | 0.0276 | No |
| 117 | ZFHX4 |  |  | 4941 | -0.017 | 0.0193 | No |
| 118 | GIGYF2 |  |  | 4989 | -0.018 | 0.0152 | No |
| 119 | NRCAM |  |  | 5003 | -0.018 | 0.0150 | No |
| 120 | MIB1 |  |  | 5106 | -0.021 | 0.0046 | No |
| 121 | RBBP4 |  |  | 5127 | -0.021 | 0.0038 | No |
| 122 | AGPAT4 |  |  | 5150 | -0.021 | 0.0028 | No |
| 123 | APC |  |  | 5342 | -0.025 | -0.0176 | No |
| 124 | PLS3 |  |  | 5389 | -0.026 | -0.0210 | No |
| 125 | PTBP2 |  |  | 5423 | -0.027 | -0.0229 | No |
| 126 | ZNF445 |  |  | 5474 | -0.028 | -0.0267 | No |
| 127 | CASP2 |  |  | 5494 | -0.028 | -0.0269 | No |
| 128 | ACOX1 |  |  | 5517 | -0.029 | -0.0274 | No |
| 129 | STAG1 |  |  | 5538 | -0.029 | -0.0276 | No |
| 130 | PPP1R12A |  |  | 5573 | -0.030 | -0.0294 | No |
| 131 | DBN1 |  |  | 5587 | -0.030 | -0.0287 | No |
| 132 | SGSM2 |  |  | 5632 | -0.031 | -0.0316 | No |
| 133 | VPS36 |  |  | 5661 | -0.032 | -0.0325 | No |
| 134 | UBE4B |  |  | 5698 | -0.032 | -0.0343 | No |
| 135 | SERINC5 |  |  | 5716 | -0.033 | -0.0340 | No |
| 136 | MEX3D |  |  | 5738 | -0.033 | -0.0340 | No |
| 137 | STAU2 |  |  | 5783 | -0.034 | -0.0366 | No |
| 138 | FOXK2 |  |  | 5948 | -0.037 | -0.0530 | No |
| 139 | VGLL4 |  |  | 6018 | -0.038 | -0.0583 | No |
| 140 | ANKRD50 |  |  | 6045 | -0.039 | -0.0585 | No |
| 141 | CCDC88A |  |  | 6086 | -0.040 | -0.0602 | No |
| 142 | PTBP3 |  |  | 6094 | -0.040 | -0.0581 | No |
| 143 | XRN1 |  |  | 6096 | -0.040 | -0.0554 | No |
| 144 | GOLGB1 |  |  | 6206 | -0.043 | -0.0649 | No |
| 145 | RWDD1 |  |  | 6239 | -0.043 | -0.0655 | No |
| 146 | SRSF2 |  |  | 6250 | -0.044 | -0.0635 | No |
| 147 | KIF2A |  |  | 6294 | -0.044 | -0.0653 | No |
| 148 | NCAM1 |  |  | 6342 | -0.046 | -0.0675 | No |
| 149 | DCLK1 |  |  | 6391 | -0.047 | -0.0697 | No |
| 150 | MAPK10 |  |  | 6491 | -0.049 | -0.0776 | No |
| 151 | PSMC6 |  |  | 6525 | -0.050 | -0.0778 | No |
| 152 | MAGI2 |  |  | 6760 | -0.058 | -0.1008 | No |
| 153 | CDC27 |  |  | 6787 | -0.058 | -0.0996 | No |
| 154 | NUP85 |  |  | 6842 | -0.060 | -0.1016 | No |
| 155 | KIAA1109 |  |  | 6913 | -0.062 | -0.1052 | No |
| 156 | TENM1 |  |  | 6954 | -0.063 | -0.1053 | No |
| 157 | ADD1 |  |  | 6959 | -0.063 | -0.1011 | No |
| 158 | CDK14 |  |  | 6970 | -0.064 | -0.0977 | No |
| 159 | PDE4D |  |  | 7047 | -0.066 | -0.1017 | No |
| 160 | TTC21B |  |  | 7081 | -0.067 | -0.1007 | No |
| 161 | EEA1 |  |  | 7137 | -0.069 | -0.1021 | No |
| 162 | CXCR4 |  |  | 7242 | -0.072 | -0.1090 | No |
| 163 | OFD1 |  |  | 7259 | -0.073 | -0.1056 | No |
| 164 | FNBP1 |  |  | 7333 | -0.075 | -0.1086 | No |
| 165 | SEMA6D |  |  | 7344 | -0.076 | -0.1043 | No |
| 166 | AKAP9 |  |  | 7436 | -0.079 | -0.1092 | No |
| 167 | ERP44 |  |  | 7572 | -0.085 | -0.1187 | No |
| 168 | NAE1 |  |  | 7583 | -0.085 | -0.1137 | No |
| 169 | ADAM10 |  |  | 7615 | -0.087 | -0.1111 | No |
| 170 | SOX6 |  |  | 7616 | -0.087 | -0.1048 | No |
| 171 | CLINT1 |  |  | 7642 | -0.088 | -0.1014 | No |
| 172 | DCAF10 |  |  | 7668 | -0.089 | -0.0978 | No |
| 173 | TSPAN3 |  |  | 7687 | -0.090 | -0.0934 | No |
| 174 | ANKRD11 |  |  | 7843 | -0.098 | -0.1043 | No |
| 175 | ST8SIA5 |  |  | 7931 | -0.103 | -0.1070 | No |
| 176 | ANK3 |  |  | 7938 | -0.103 | -0.1002 | No |
| 177 | PAM |  |  | 8068 | -0.111 | -0.1072 | No |
| 178 | SEMA6A |  |  | 8098 | -0.113 | -0.1023 | No |
| 179 | BMPR2 |  |  | 8138 | -0.117 | -0.0984 | No |
| 180 | NETO2 |  |  | 8164 | -0.118 | -0.0928 | No |
| 181 | PSMB1 |  |  | 8169 | -0.119 | -0.0847 | No |
| 182 | GRIA1 |  |  | 8237 | -0.124 | -0.0835 | No |
| 183 | RNMT |  |  | 8277 | -0.129 | -0.0787 | No |
| 184 | TPRKB |  |  | 8438 | -0.151 | -0.0864 | No |
| 185 | TMEM106B |  |  | 8450 | -0.152 | -0.0766 | No |
| 186 | ATXN1 |  |  | 8456 | -0.153 | -0.0662 | No |
| 187 | MIR181A1HG |  |  | 8461 | -0.154 | -0.0555 | No |
| 188 | MSRA |  |  | 8546 | -0.172 | -0.0529 | No |
| 189 | KIF20B |  |  | 8576 | -0.180 | -0.0432 | No |
| 190 | TTC14 |  |  | 8635 | -0.205 | -0.0351 | No |
| 191 | DST |  |  | 8644 | -0.211 | -0.0208 | No |
| 192 | TMEM59 |  |  | 8723 | -0.261 | -0.0110 | No |
| 193 | FUS |  |  | 8731 | -0.268 | 0.0076 | No |
Table: GSEA details [plain text format]

  

Fig 2: RICHARDS\_NOWAKOWSKI\_SCIENCE\_2017\_IN\_CTX\_MGE2\_UPREG: Random ES distribution      
 Gene set null distribution of ES for **RICHARDS\_NOWAKOWSKI\_SCIENCE\_2017\_IN\_CTX\_MGE2\_UPREG**
